# Incidence and factors associated with flare-ups in a post graduate programme in the indian population

**DOI:** 10.4317/jced.51578

**Published:** 2014-12-01

**Authors:** Jaya Pamboo, Manoj-Kumar Hans, Bangalore-Niranjan Kumaraswamy, Subhash Chander, Sajeev Bhaskaran

**Affiliations:** 1 Post Graduate Student. Department of Conservative Dentistry and Endodontics, Vyas Dental College and Hospital, Jodhpur, Rajasthan, India; 2 Reader. Department of Conservative Dentistry and Endodontics, Vyas Dental College and Hospital, Jodhpur, Rajasthan, India; 3 Professor. Department of Conservative Dentistry and Endodontics, Vyas Dental College and Hospital, Jodhpur, Rajasthan, India; 4 Assistant Professor. Department of Conservative Dentistry and Endodontics, Vyas Dental College and Hospital, Jodhpur, Rajasthan, India; 5 Professor and Head. Department of Conservative Dentistry and Endodontics, Vyas Dental College and Hospital, Jodhpur, Rajasthan, India

## Abstract

Objectives: The study had twin objectives: to assess the incidence of flare-ups (a severe problem requiring an unscheduled visit and treatment) among patients who received endodontic treatment in the Department of Conservative Dentistry and Endodontics in Vyas Dental college and hospital, Jodhpur during a period of one year, and also to examine the correlation with pre-operative and operative variables. Material and Methods: Data was collected from 1023 teeth from 916 patients who had received endodontic treatment over a 12- month period. Information was obtained for each patient treated, including pulp and peri-radicular diagnosis for the tooth, presence of pre-operatory pain, type of medication being used, type of instrumentation technique used and number of appointments needed to complete the root canal treatment. Results: The results showed an incidence of 2.35% for flare-ups from 1023 endodontically treated teeth. Statistical analysis was done using the chi-square test. Conclusions: Flare-ups were found to be affected significantly by gender of patient, presence of radiolucent lesions, patients taking pre-operative analgesic or anti-inflammatory drugs and on type of instrumentation technique. In contrast, there was no correlation between flare-ups and age, different arch/tooth groups and single or multiple visit endodontics.

** Key words:**Anti-inflammatory, flare-ups, instrumentation, prospective.

## Introduction

Flare-up is a commonly used term with the characteristic symptoms of pain and swelling arising following endodontic treatment with its best treatment solution being its prevention of occurrence (1). Mechanical, chemical and/or microbial injury to the pulp or periradicular tissues are considered to be the causative factors of flare-ups (1).

A flare-up may be defined as the occurence of severe pain and/or swelling following an endodontic treatment appointment, requiring an unscheduled visit for treatment. It is a well-known complication that disturbs both patients and dentists (2). Endodontic Inter appointment Emergency [EIE] is another term used for flare-up (3). The aetiological factors, can be divided into three main areas: 1: treatment routine and clinical procedures under the control of the operator (3); 2: microbial factors related to the contents of the infected root canals and 3: host factors such as patient demographics [gender, age and tooth group], local tissue changes, immunological phenomena, and various psychological factors (3). Flare-ups have been correlated with variables such as the age of the patient, gender, different arch and tooth groups, pulp/periradicular diagnosis, presence of preoperative signs and symptoms, systemic conditions, operator skill and treatment modalities that include patients on medication before and during the clinical procedures, type of treatment [conventional or rotary], number of appointments, complete/incomplete debridement of the canals, over-instrumetation, and level of the root fillng (3).

Flare-ups prevalence is reported as low as 0.39% (4), to as high as 20 % (5) which can be explained by different temperament of flare up (2). It is described as pain and/or swelling that requires active interference by a dentist while others prefer to use a quantitative method with index questionnaire to describe this phenomenon (6). Pushing microorganisms beyond the apex is considered the most significant cause of flare-ups (7). Preoperative pain, anxiety, type of intracanal medicine and size of apical lesion are considered as causative factors affecting the rate of flare ups (8-12). Due to changes in definitions, concepts and materials in endodontics, study in this field seems to be necessary (2). A prospective study involving a large number of teeth could clarify the factors related to the presence of post treatment sequelae. Therefore, the purpose of this study was to examine the overall incidence of flare-ups as a percentage of the total endodontic treatments performed during a period of a year, and also to correlate it with; 1: patient demographics [age, sex and tooth group/arch]; 2: tooth diagnosis [pulp/periradicular Diagnosis; and 3: treatment factors [patients receiving medication, type of treatment and number of appointments].

## Material and Methods

Data was collected from 1049 teeth from 936 patients who had received endodontic treatment over a 12- month period in the Department of Conservative and Endodontics in Vyas Dental College and Hospital, Jodhpur by three different operators. Informed consent was taken from all the participants and approval from institutional ethical committee was taken. Information was obtained for each patient treated, including pulp and peri-radicular diagnosis for the tooth, presence of pre-operatory pain, type of medication being used, type of instrumentation technique used and number of appointments needed to complete the root canal treatment.

Inclusion critera included patients aged between 15 to 66 years, average age of 42 years, teeth with fully formed roots, teeth with non-calcified canals, teeth with normal pulp for intentional endodontic treatment, teeth with irreversible pulpitis, teeth with apical periodontitis with and without any periapical lesion.

Exclusion criteria included retreatment cases, acute pain with swelling cases, teeth with mobility, pregnant patients.

Under rubber dam isolation, conventional straight-line access preparations were made and a step-back instrumentation technique was used as the routine procedure in teeth treated using hand filling in which the coronal two-thirds of the root canal was enlarged with Gates Glidden burs [Dentsply Maillefer, Ballaigues, Switzerland], sizes 070 and 090, The working length was established 1 mm from the radiographic apex. The apical third was then prepared using K files [K endo, VDW, Germany] with step-back increments of 0.5 mm until a final file size 30 to 45 could be placed at the working length. The criteria used to determine the end of the preparation were: (1) an apical stop was present; and (2) a fine finger spreader penetrated close [1-2 mm] to the working length, irrigation was copious and frequent: 1ml of 5.25% sodium hypochlorite was delivered from a 30-gauge needle [Unolock, Hindustan syringes] after each file change. A final irrigation was performed using chlorhexidine for 1 min in each canal. In the cases treated with rotary files, Protaper [Dentsply Maillefer, Ballaigues, Switzerland] rotary files were used in crown-down instrumentation technique till F3 in distal canals of lower molars, palatal canals of upper molars and buccal and lingual canals two rooted premolars and till F2 in mesial and distal canals of upper and lower molars and till F4 in single rooted premolars. All hand filing cases were obturated with gutta percha points [Tanari, Manacapuru, AM, Brazil] and AH plus sealer [Dentsply Maillefer, Ballaigues, Switzerland] using the lateral condensation technique and in rotary filing cases obturation was done using single cone Gutta percha and AH plus sealer. Some cases were completed in one appointment, although some, as a result of many factors such as abscess, fatigue of the patient, or lack of time and complexity of a particular case, needed additional appointments. In these cases, root canals were dried and calcium hydroxide [Calen, SS White, Rio de Janeiro, Brazil] was used as an intracanal medicament and access cavity was temporarily sealed with IRM cement [Dentsply De Trey GmbH, Konstanz, Germany]. After each appointment, the patient received careful verbal instructions as follows: After endodontic therapy, the treated tooth is expected to be sensitive, especially when chewing. This is considered normal and over-the-counter analgesics usually alleviate this discomfort. If patient experiences spontaneous pain, particularly if tooth pain is getting worse and/or swelling is developing or increasing within a week, please call the doctor. In such cases where severe pain and/or swelling developed, an emergency visit was arranged and active treatment was carried out as deemed appropriate, using either reinstrumentation of the canal system [multiple-visit cases], surgical drainage [one-visit cases] or both. At this time, the patient’s case notes were retrieved and classified as a flare-up. The overall incidence of flare-ups was recorded and expressed as a percentage of the total number of teeth evaluated. The percentage of flare-ups related to the studied variables was also determined. These percentages were statistically compared using the chi-square test and Multivariate logistic regression analysis is done to determine the influence of the pre-operative diagnosis on the flare-up incidence.

## Results

Out of total 936 patients, twenty patients did not participated in the study as they did not turn up after first appointment and so total 916 patients were included in the study from which 1023 teeth were examined and twenty four required emergency treatment, resulting in an overall incidence of flare-ups of 2.35%. No statistically significant difference was found for age or for different arch and tooth groups but a significant difference was found for gender ( Table 1) with highest flare up occurring in females than males. On the other hand, periradicular diagnosis was positively related to flare-ups ( Table 2), the presence of a lesion resulted in a statistically significant increase of emergencies when compared to teeth which showed a radiographically normal appearance of the periapical region. The variables concerned with treatment factors were statistically analysed and showed a significant difference among patients on medication, with the highest incidence of flare-ups found in patients without any analgesics/anti-inflammatory drugs and antibiotics and also No significant difference was found in the number of visits [single as compared to multiple] but a significant difference was noted when a comparison was made between teeth treated with rotary files and hand files. More flare ups were found in hand files then rotary files ( Table 3). Multivariate logistic regression analysis done using the incidence of flare-up as dependent variable and to determine the influence of the pre-operative diagnosis on the flare-up incidence ( Table 4) showed that as the value of coefficient is negative so the occurrence of flare up is very less for Irreversible Pulpitis than for Apical Periodontitis than for Normal and it is highest for Apical periodontitis with Lesion present.

**Table 1:**
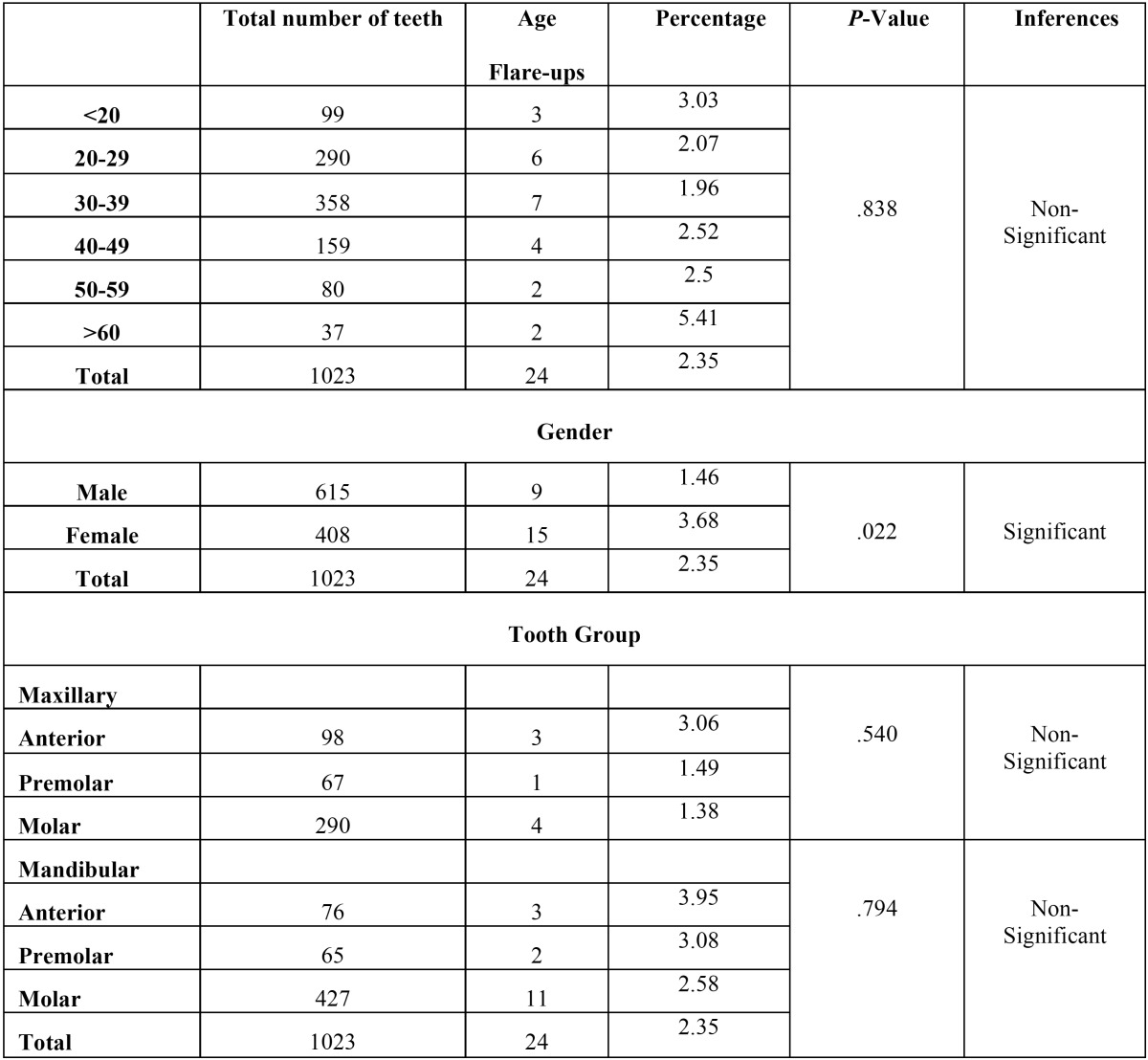
Occurrence of flare-ups in different age groups, gender and tooth groups.

**Table 2:**
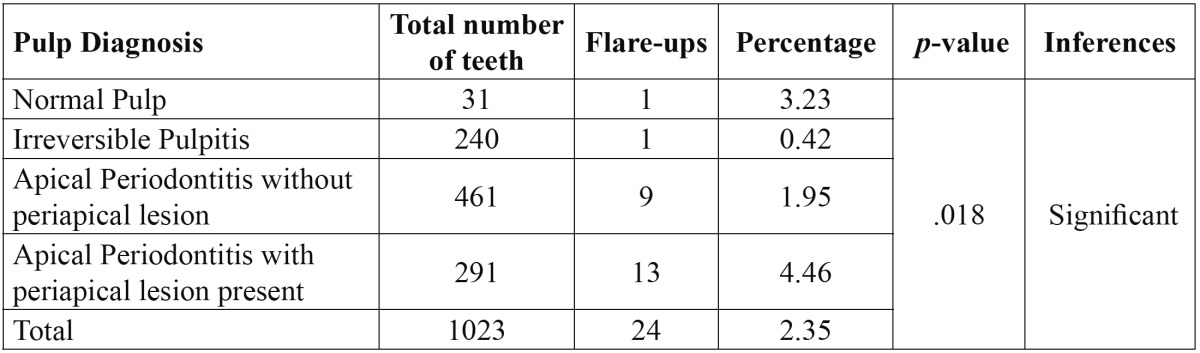
Occurrence of flare-ups related to pulp diagnosis.

**Table 3:**
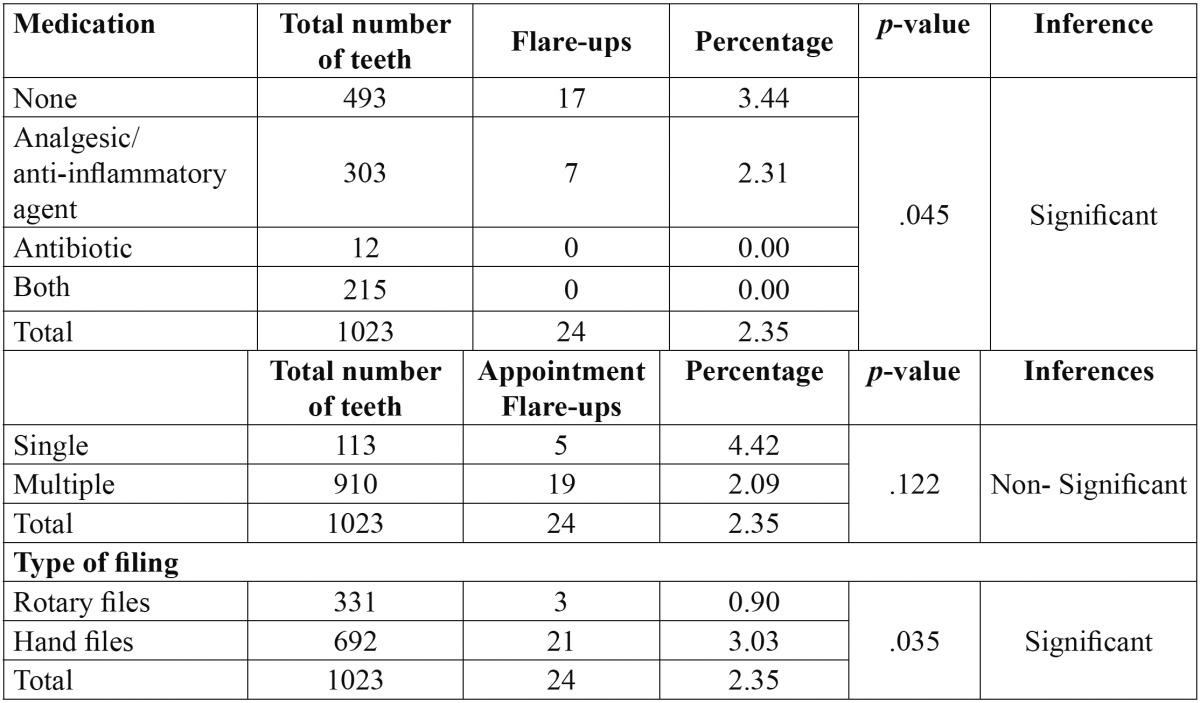
Occurrence of flare-ups in patients on medication Occurrence of flare-ups according to the number of appointments and type of filing.

**Table 4:**
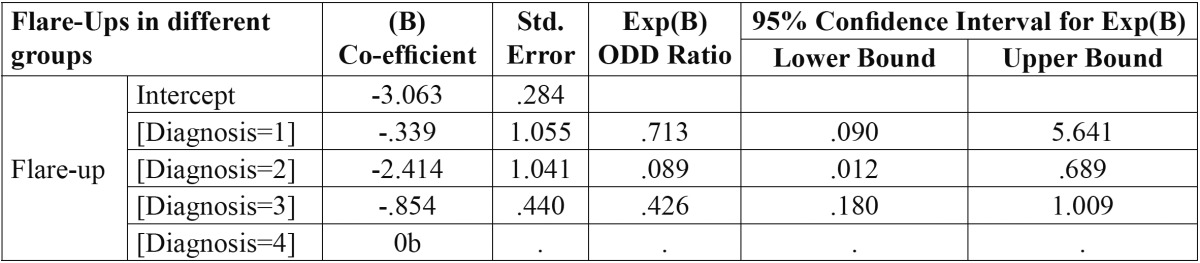
Multivariate logistic regression analysis using the incidence of flare-up as dependent variable and to determine the influence of the pre-operative diagnosis on the flare-up incidence.

## Discussion

This study showed a low incidence of flare-ups following endodontic treatment [2.35%]. These results can be compared to studies who also reported similar findings (2,9,10). To evaluate flare-ups, criterias used were, occurrence of severe spontaneous pain and/or swelling developing after root canal treatment requiring an unscheduled visit for treatment (10). Root canal therapy is considered to be a routine dental procedure and it is in variance with the belief of many patients that endodontic treatment is painful. Such widespread apprehensions causes unnecessary anxiety and fear (11). Patient’s pain perception and threshold of reaction are influenced by fear of dentists and dental procedures, anxiety, apprehension, and many other psychological factors (11). It has been observed from these studies that root canal treatment conducted under sound biological principles, using scientifically based techniques, a low incidence of flare-ups are expected (9,10).

An analysis of the influence of patient age or tooth/arch groups with flare-up occurrence, showed no statistical significant difference. These findings are in agreement with several authors (2,10). Studies have failed to find any evidence indicating that age is a risk factor for development of flare-ups (12).

In contrast, other studies have found that treatment outcome levels significantly decreases with increasing age (8) as well as patients older than 35 years feels less pain compared to patients aging 35 years and younger (8). Since there is no data which states that progressive loss of sensitivity to nociceptive stimuli occurs with age (13) hence age-related decrease in pain is not attributed to changes in the physiological pain system (14).

Gender differences in pain states that women reports more pain than men and also the reduction in pain thres-holds (15). Females are more likely to seek treatment when they experience significant symptoms (16). This has been consistently demonstrated in investigations which compares males behaviour with females, when pain is a factor (12).

Studies determining the prevalence of persistent pain following endodontic treatment, concludes that females are more associated with persistent pain after successful endodontic treatment (17). Higher levels of allodynia, defined as reduced mechanical pain thresholds were found in women with irreversible pulpitis and acute periradicular periodontitis, compared to men (18).

Higher levels of post-endodontic pain have been reportedly associated significantly with posterior teeth in the mandibular arch (19). Incisors and canines in comparison to premolars and molars have shown significant differences in pain levels in between treatments (20). High frequency of more number of canals and bifurcated root canals in posterior teeth are related to this difference (20). The duration of the treatment being longer in molar teeth, could also explain this result, taking into account the progressive decrease of the anaesthetic effect, along with the increase of the anxiety of the patient as the intervention extended. Previous study has showed that the percentage of patients not feeling pain decreases as the duration of the procedure increases (20). However, other studies have not found differences in pain level in relation to tooth type (21).

The incidence of flare-up is having direct relationship with the severity of the patient’s preoperative diagnosis and signs and symptoms. The incidence of flare-ups is higher with necrotic pulp tooth than in vital tooth. Studies have found the incidence of flare-ups in tooth with necrotic pulp being 7.17% (22). Establishing accurate working length of tooth and complete instrumentation of root canal in the first appointment is the best method of managing the necrotic pulp. Removal of debris from the canal should be the main goal (22).

Antibiotic usage generally shows low incidence of flare up. Majority of cases of pain showed its resolution within 48 hours. Hence it can be concluded that most of the postoperative pain can be easily controlled by an-ti-inflammatory medication (23). Antibiotics can be judiciously used to manage a flare-up. Some patients are prophylactically placed on an antibiotic to reduce the potential for a flare-up (24). Clinicians must use antibiotics appropriately. Non-steroidal anti-inflammatory drugs [NSAID] are potent anti-inflammatory agents and are helpful in reduction of swelling and pain (24).

Present study reported no significant difference in flare ups between single visit and multiple visit treatment. Another unusual finding was that postoperative pain frequency for patients treated in one appointment was higher [16%] than patients treated in two appointments [9.6%] (25). Most other studies have found that one-visit treatments resulted in less pain than those taking two visits (26).

Root canal instrumentation using step-back technique with hand files produced significantly more pain perception than rotary files. The engine driven techniques extrude smaller amounts of debris and irrigants, presumably due to the rotary motion, which tends to direct debris toward the orifice, avoiding its compactation in the root canal (27). However, in hand filing, the filing action of the instrument pumps the irrigation solution and debris through the apex (28). Since exclusively using clockwise or alternate rotary motions, debris is extruded beyond the apical foramen and it is considered as one of main responsible factors of flare-up. Studying postobturation pain of different origin after endodontic treatment, reported that “crown down” preparation using completely rotating instruments proved to be effective as regards prevention of postoperative pain (29). The effects of the technique used for root canal instrumentation on emergence of pain after endodontic therapy have been analyzed (30) and it was found that the least risk of pain emergence after endodontic treatment occurs with tooth canal widening by crown-down technique.

Flare-up can be prevented by selection of an instrumentation technique that extrude less amount of debris apically, which usually is the crown down technique with rotary action combined with copious and frequent irrigation. Intracanal medication is a preventive measure that is used to reduce the incidence of inter-appointment flare-ups. But still more comparative studies are required, to find out the best treatment option available for the prevention of flare-up.

## Conclusions

In this study it was concluded that flare-ups were found to be positively correlated with gender of patient, presence of radiolucent lesions, patients taking analgesic or anti-inflammatory drugs and on type of instrumentation technique. In contrast, there was no correlation between flare-up, and age, different arch/tooth groups and single or multiple visit endodontics.
